# Mechanistic and structural studies of KDM‐catalysed demethylation of histone 1 isotype 4 at lysine 26

**DOI:** 10.1002/1873-3468.13231

**Published:** 2018-09-14

**Authors:** Louise J. Walport, Richard J. Hopkinson, Rasheduzzaman Chowdhury, Yijia Zhang, Joanna Bonnici, Rachel Schiller, Akane Kawamura, Christopher J. Schofield

**Affiliations:** ^1^ Department of Chemistry Chemistry Research Laboratory University of Oxford UK; ^2^ Leicester Institute of Structural and Chemical Biology and Department of Chemistry University of Leicester UK; ^3^ Division of Cardiovascular Medicine Radcliffe Department of Medicine The Wellcome Trust Centre for Human Genetics Oxford UK

**Keywords:** 2‐oxoglutarate oxygenases, demethylases, epigenetics, histones, JmjC demethylases, lysine *N*‐methylation

## Abstract

*N*‐Methylation of lysyl residues is widely observed on histone proteins. Using isolated enzymes, we report mechanistic and structural studies on histone lysine demethylase (KDM)‐catalysed demethylation of *N* ^ε^‐methylated lysine 26 on histone 1 isotype 4 (H1.4). The results reveal that methylated H1.4K26 is a substrate for all members of the KDM4 subfamily and that KDM4A‐catalysed demethylation of H1.4K26me3 peptide is similarly efficient to that of H3K9me3. Crystallographic studies of an H1.4K26me3:KDM4A complex reveal a conserved binding geometry to that of H3K9me3. In the light of the high activity of the KDM4s on this mark, our results suggest JmjC KDM‐catalysed demethylation of H1.4K26 may be as prevalent as demethylation on the H3 tail and warrants further investigation in cells.

## Abbreviations

**2OG**, 2‐oxoglutarate

**FDH**, formaldehyde dehydrogenase

**HP1**, heterochromatin protein 1

**KDM**, histone lysine demethylase

**KMT**, S‐adenosylmethionine‐dependent methyltransferase

**NOG**, *N*‐oxalylglycine

Widespread *N*‐methylation of both arginyl and lysyl residues occurs on the tails of core histones (histones H2A, H2B, H3 and H4), where it has roles in regulating gene expression 1. Lysyl residues can undergo successive methylations on the *N* ^ε^‐nitrogen to give three methylation states (mono‐*N* ^ε^‐methyllysine, di‐*N* ^ε^‐methyllysine and tri‐*N* ^ε^‐methyllysine respectively), as catalysed by *S*‐adenosylmethionine‐dependent methyltransferases (KMTs) 2, 3. In many cases, KMTs catalyse methylation at multiple sites to give different methylation states, on both histones and non‐histone proteins 1, 4. Enzyme‐catalysed demethylation of methylated lysyl residues also occurs at many sites on histones H3 and H4 1. Thus, at least at certain sites, lysyl methylation is dynamically regulated by the interplay between the two enzyme‐catalysed processes.

**Figure 1:**
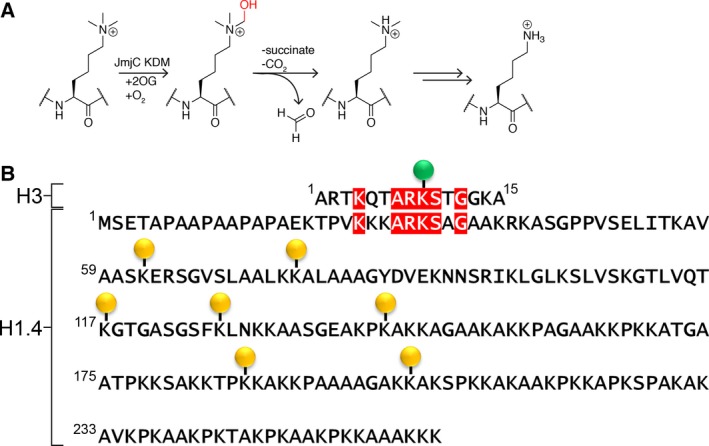
Demethylation by the JmjC KDMs. (A) Outline mechanism of histone lysine demethylation as catalysed by the JmjC KDMs. (B) Sequence alignment of the first 15 residues of histone H3 with the sequence of histone H1.4. The H3K9 position known to be methylated is marked with a green circle. Other positions known to be methylated on H1.4 are marked with yellow circles 12, 13, 45.

Demethylation is catalysed by two families of histone lysine demethylases (KDMs) 5. Members of the larger family of KDMs, JmjC KDMs, catalyse methyl group oxidation coupled to oxygen‐dependent oxidative decarboxylation of 2‐oxoglutarate (2OG), forming succinate and carbon dioxide (Fig. 1A) 5. The available evidence implies that the resultant hemiaminal is normally unstable, fragmenting to give the demethylated lysyl residue and formaldehyde 6. Dysregulation of JmjC KDM family members is implicated in many diseases, including developmental disorders and many cancers. The JmjC KDMs are thus the focus of drug development programmes 7, although their physiological roles are, in many cases, incompletely defined. There is emerging evidence that certain KDMs have multiple substrates including non‐histone proteins and potentially methylated arginyl residues 8, 9, suggesting the possibility for *in vivo* pleiotropy in KDM catalysis.

Though less well studied, lysyl methylation has been observed on histones other than H3 and H4. Histone 1 isotype 4 (H1.4), as opposed to the core histones H2A, H2B, H3 and H4, is one of multiple ‘linker’ histones that bind to nucleosomal DNA, but which do not form part of the histone octamer 10. Linker histones have been traditionally viewed as playing important structural roles in stabilising higher order chromatin structure 11. More recent evidence suggests that histone 1 isotypes and their post‐translational modifications also have additional functional roles within the cell, including in transcriptional regulation 11, 12. To date, a number of sites of histone H1.4 lysyl *N* ^ε^‐methylation have been identified (Fig 1B), with H1.4K26 being the most abundantly methylated site on H1.4 13. The methylation status of H1.4K26 is reported to vary dynamically during the cell cycle 14, and to undergo KDM4‐catalysed demethylation, as supported by *in vitro* studies on mouse KDM enzymes 15. The methylation status of the H1.4K26 residue is commonly associated with repressed genes, and is known to regulate binding of the family of heterochromatin protein 1 (HP1) proteins and related chromodomains, such as CDYL2 16, 17. Loss of H1.4K26 (by substitution with a histone H1.4 variant bearing an alanine residue at this position, H1.4K26A) results in a reduction of cell proliferation, deregulation of gene expression and a reduction of stabilisation of H1.4 in heterochromatic regions 14. Methylation of H1.4K26 also enhances phosphorylation of H1.4K27 by the Aurora B kinase, which in turn hinders recruitment of HP1 to H1.4K26me2 17, 18. H1.4K26 is also reported to be acetylated by an unknown acetyltransferase, and to be deacetylated by SIRT1 19. Thus, emerging, and incompletely characterised, results indicate that complex regulatory loops exist for H1.4, in addition to those established with the core histones.

All human KDM4 enzymes catalyse demethylation of H3K9 methylation, with KDM4A‐C additionally catalysing H3K36 demethylation 20, 21, 22, 23. Some KDM4 subfamily members have also been demonstrated to catalyse *in vitro* demethylation of *N* ^ε^‐methylated H3K27 and arginine residues in the H3 and H4 tails 8, 24. With respect to their activity on H1.4, it is currently unclear (a) how the demethylation efficiency at H1.4K26 compares to other KDM4 substrates (H3K9 and H3K36), (b) how the H1.4K26 substrate binds within KDM4 active sites, and also (c) whether JmjC KDMs other than the KDM4s can catalyse demethylation at H1.4K26.

To address these questions, we report studies investigating human JmjC KDM‐catalysed demethylation of H1.4K26 methylation. Our findings reveal that demethylation of methylated lysyl residues in H1.4K26 peptide fragments is catalysed by all members of the human KDM4 subfamily of JmjC KDMs (KDM4A‐E), supporting previously reported cell‐based studies (predominantly with mouse enzymes) 15. Unexpectedly, we also observed low level H1.4K26me2 demethylation activity by KDM7A and KDM7B (PHF8), in the context of fragment peptides, though at levels that may not be biologically relevant. While demethylation of H1.4K26me2/1‐containing peptides is relatively inefficient for KDM7A and KDM7B, kinetic analyses indicate that H1.4K26me3 is a comparably efficient substrate for KDM4A as H3K9me3 (at least in the context of fragment peptides) and is a better substrate than H3K36me3. This assignment is supported by crystallographic studies indicating that the H1.4K26me3 peptide binds to the KDM4 active site in a similar manner to the H3K9me3 peptide. Overall, H1.4K26me3 appears to be a good substrate for KDM4A, and indeed other KDM4 enzymes, which is supportive of its assignment as a KDM4 substrate in mouse and human cells 15.

## Materials and methods

### Protein production

The catalytic domains of the JmjC KDMs used in this work were recombinantly expressed as reported either in *Escherichia coli* (KDM2A_1–557_ 25, KDM4A_1–359_ 26, KDM4B_1–359_, KDM4C_1–359_, KDM4D_1–359_, KDM4E_1–337_ 27, KDM6B_1141–1590_ 28, KDM7B_1–447_ 29, KDM7A_38–480_ 28) or Sf9 cells (KDM3A_515–1317_ 28, KDM5C_1–765_ 28) as N‐terminally His6‐tagged proteins. Proteins were purified by Ni‐affinity chromatography followed by size exclusion chromatography as previously described.

### Peptide synthesis

Peptides were produced as C‐terminal amides using a Liberty Blue automated microwave peptide synthesiser (CEM Corporation, Matthews, NC, USA), using standard fluorenylmethyloxycarbonyl‐mediated solid‐phase chemistry and NovaPEG rink amide resin (Merck, Kenilworth, NJ, USA). Following synthesis, peptides were cleaved from the resin by incubation with trifluoroacetic acid/water/triisopropylsilane/dimethoxybenzene (92.5 : 2.5 : 2.5 : 2.5) for 3 h followed by precipitation with ice‐cold diethyl ether. For kinetic experiments, lyophilised peptides were purified by reverse‐phase high‐performance liquid chromatography using a Vydac C18 column (Solvent A: 0.1% trifluoroacetic acid in H_2_O, Solvent B: 0.1% trifluoroacetic acid in acetonitrile). Sequences are given in Table S1.

### MALDI‐TOF MS activity assays

Recombinant proteins (1 μm) were incubated with peptides (10 μm, sequences are given in Table S1) in 50 mm HEPES, pH 7.5 with addition of Fe(II) ammonium sulfate (10 μm), sodium ascorbate (100 μm) and 2‐oxoglutaric acid (200 μm). Reactions with KDM3A and KDM7A also contained 1 mm tris(2‐carboxyethyl)phosphine hydrochloride. No enzyme reactions were included as negative controls. Reactions were incubated for 1 h at 37 °C before being quenched with 1 : 1 (v/v) methanol. Analysis was carried out by MALDI‐TOF MS (Bruker, Billerica, MA, USA), with demethylation observed as mass shifts of 14 Da.

### NMR activity assays

The NMR analyses employed a Bruker Avance III 700 MHz spectrometer with an inverse TCI cryoprobe optimised for ^1^H observation and were carried out as previously described 6. Samples (KDM4A (10 μm), 2OG (400 μm), H1.4(18–32)K26me3 peptide (400 μm), Fe(II) ammonium sulfate (100 μm) and ascorbate (1 mm) in 50 mm ammonium formate, pH 7.5, 500 mm NaCl in 10% v/v D_2_O) were prepared in a microcentrifuge tube (total volume = 75 μL), before being transferred to an NMR tube. The samples were then analysed over time using automated routines; each time‐point corresponded to one experiment accumulating 16 transients (89 s total acquisition time per experiment). The solvent resonance was depleted by excitation sculpting using a 180° sinc pulse (duration = 2 ms) 30.

### Kinetic analyses

Kinetic parameters for KDM4 enzymes were determined by use of an FDH (formaldehyde dehydrogenase)/NAD^+^‐coupled) assay for quantification of the formaldehyde reaction byproduct, as previously described 27. Assays were carried out in 50 mm HEPES, pH 7.5, 0.01% Tween‐20, with addition of Fe(II) ammonium sulfate (10 μm), sodium ascorbate (100 μm), 2‐oxoglutarate (200 μm), NAD^+^ (500 μm), KDM4 enzymes (1 μm for specific activities, 500 nm for Michaelis–Menten kinetics) and FDH enzyme (0.001 U·μL^−1^, Sigma‐Aldrich, St Louis, MO, USA) in a 30‐μL volume in black 384‐well plates. Specific activities were measured using a 100‐μm peptide. For Michaelis–Menten experiments, peptide concentrations were varied. Reactions were monitored using a PHERAstar FS plate reader (BMG Labtech, Ortenberg, Germany) with 355 nm excitation and 460 nm emission. Kinetic parameters were calculated from the reaction rate during the initial linear phase of formaldehyde production, which were used to calculate specific activities or fitted to Michaelis–Menten equations using graphpad prism (GraphPad Software, La Jolla, CA, USA).

### X‐ray crystallography

Co‐crystals of KDM4A_1–359_.Ni(II).H1.4(18–32)K26me3.NOG were obtained in sitting drops grown at 4 °C with a ratio of 1 : 2 sample to well solution [0.1 m MIB buffer (malonic acid/imidazole/boric acid, pH 6.0, 25% w/v PEG 1500)] 26. Drops contained 10 mg·mL^−1^ KDM4A, 5 mm H1.4(18–32)K26me3, 5 mm NOG and 4 mm NiCl_2_. Crystals were cryoprotected with 25% glycerol then flash‐frozen in liquid N_2_. Data were collected using a single crystal at 100 K at the Diamond I04‐1 MX beam line and were processed with HKL2000 31. The structure was solved by molecular replacement using PHASER 32 (search model: PDB ID http://www.rcsb.org/pdb/search/structidSearch.do?structureId=2OX0) and was refined by alternative cycles of CNS 33 and PHENIX^34^, with iterative rebuilding of the refined model using COOT 35. All residues were in the allowed regions of the Ramachandran plot as calculated by PROCHECK 36. Data collection and refinement statistics are given in Table S2. The crystal structure has been deposited under PDB accession code http://www.rcsb.org/pdb/search/structidSearch.do?structureId=6H8P.

## Results

**Figure 2:**
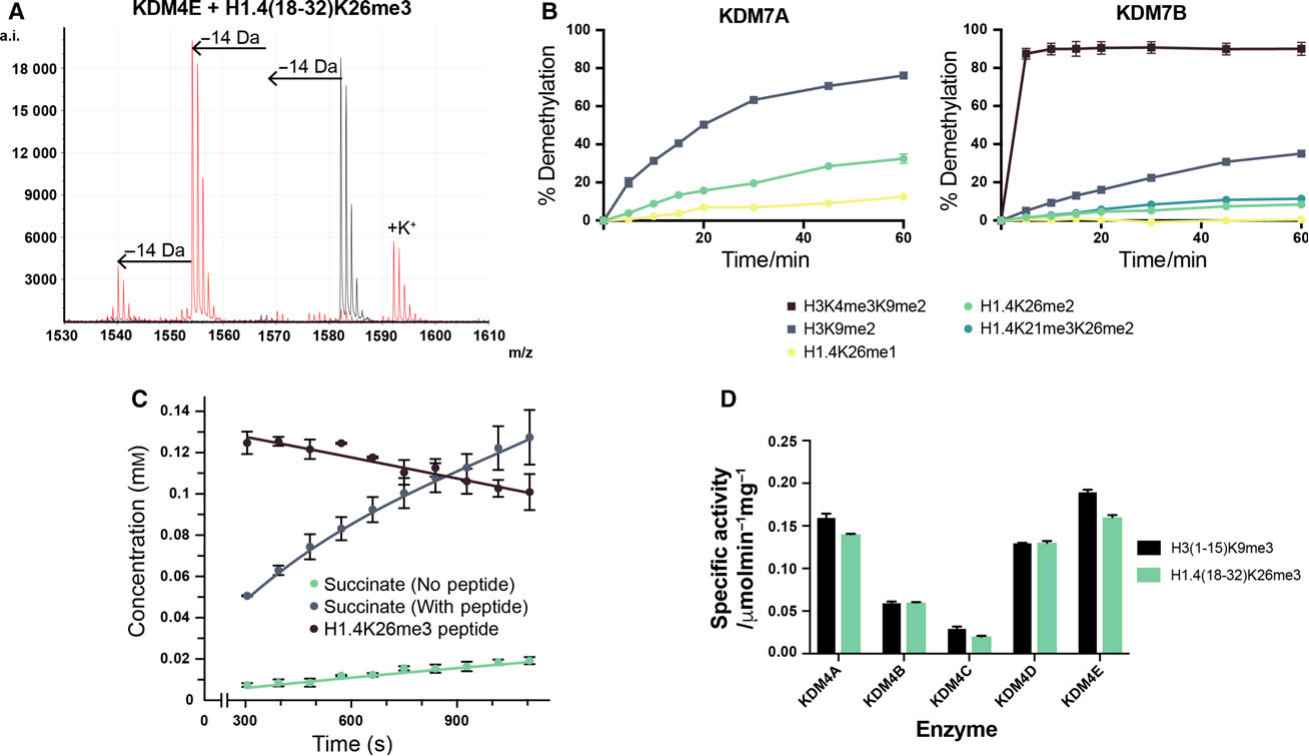
H1.4K26 N‐terminal demethylation is catalysed by JmjC KDMs. (A) Demethylation of an H1.4(18–32)K26me3 peptide by KDM4E. The red spectrum shows reactions containing enzyme; the black spectrum is a no enzyme control. (B) Comparison of activity of KDM7A and KDM7B on H1.4K26 and H3K9 methylated peptides. Time course experiments containing the stated enzyme (5 μm) and peptides (10 μm) were carried out at 37 °C, with samples removed and quenched at 8 time‐points. Samples were analysed by MALDI‐TOF MS. (C) Graphs showing the degree of succinate production and peptide demethylation of H1.4(18–32)K26me3 catalysed by KDM4A as quantified by ^1^H NMR (700 MHz). (D) Comparison of specific activity of KDM4 enzymes (1 μm) with H3(1–15)K9me3 and H1.4(18–32)K26me3 (100 μm). All data show the mean and standard deviation of technical triplicates.

The propensity of human JmjC KDMs to catalyse demethylation at H1.4K26 was investigated using recombinant forms of the catalytic domains of representatives of each human JmjC KDM subfamily (KDM2‐7) and fragment peptides (Table S1) 37. Three peptides with identical sequences, corresponding to residues 18–32 of histone H1.4 (H_2_N‐TPVKKKARKSAGAAK‐CONH_2_), but containing either a *N* ^ε^‐tri‐, di‐ or monomethyllysine at K26 were synthesised (Fig. 1B and Table S1). Activity assays monitoring JmjC KDM‐catalysed demethylation of the peptides were then carried out; samples were prepared containing each peptide, 2‐oxoglutarate (2OG), ferrous iron, ascorbate and individual representatives from each JmjC KDM subfamily (KDM2A 38, KDM3A 21, KDM4E 22, KDM5C 39, 40, KDM6B 41, KDM7A 42). After incubation for 1 h at 37 °C, matrix‐assisted laser desorption ionisation mass spectrometry (MALDI‐TOF MS) analysis revealed clear demethylation of all three H1.4 peptides containing *N* ^ε^‐lysine methylation at K26 in samples containing KDM4E, consistent with previous *in vitro* and in cell studies with closely related subfamily member, KDM4D (Fig. 2A, Table 1 and Fig. S2) 15, 43. While no evidence for demethylation was observed in samples of any of the H1.4 peptides with KDM2A, KDM3A, KDM5C or KDM6B (Table 1, Figs S3–S6), low levels of demethylation (<10%) of the mono‐ and dimethylated H1.4K26 peptides were also observed in samples containing KDM7A (Table 1, Fig. S7).

**Table 1:**
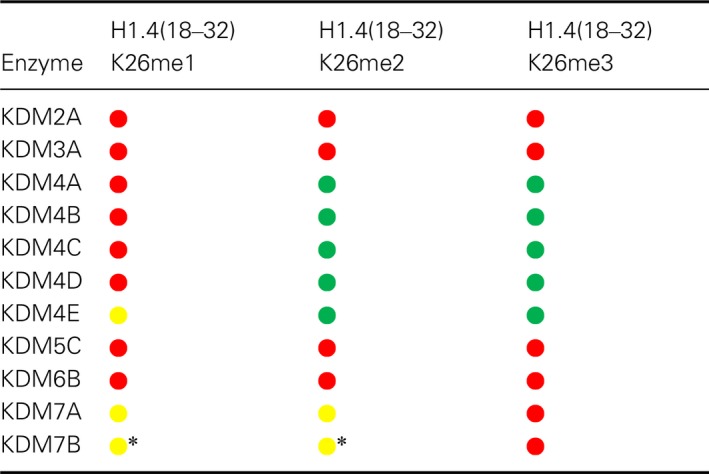
Summary of activity results for recombinant human JmjC KDMs on methylated H1.4 peptides. 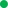 Indicates that clear demethylation activity was observed; 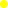 Indicates that low level demethylation activity was observed (<10% conversion after 1 h); 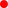 Indicates no activity under the stated conditions (1 μm KDM, 10 μm peptide); *Indicates demethylation activity was only observed with 5 μm KDM

Given the activity observed with KDM4E and KDM7A, we decided to investigate the intra‐subfamily conservation of this demethylation activity. The demethylation activity of the other subfamily members (KDM4A‐D and KDM7B) was therefore screened. For all KDM4 subfamily members, we observed H1.4K26 demethylation activity similar to that already observed with KDM4E (Table 1, Figs S8–S11); however, no H1.4K26 demethylation activity was observed with KDM7B at this enzyme concentration (Fig. S12). Note that as with H3K9, demethylation of the monomethylated H1.4K26 peptide was only observed with KDM4E, under the stated conditions 24.

*N* ^ε^‐Trimethylated H3K4 binds to the plant homeobox domain of KDM7B, targeting the *N* ^ε^‐dimethyllysine at H3K9 to the catalytic domain, and thus promoting demethylation 29, 44. To test whether similar targeting may occur with H1.4, promoting activity at H1.4K26, an H1.4 peptide was synthesised containing *di‐N* ^ε^‐methyllysine at K26 and tri‐*N* ^ε^‐methyllysine at K21 (i.e. at the equivalent position in the sequence to K4 in the H3 peptide, Fig. 1B, Table S1). Monomethylation at H1.4K21 has previously been observed by proteomic mass spectrometry 45. With the aim of enhancing activity, the enzyme concentration was increased from 1 μm to 5 μm, and time course analyses were conducted with both KDM7A and KDM7B, both with the new peptide (H1.4(18–32)K21me3K26me2) and the H1.4 peptides methylated only at K26 (Fig. 2B). At this increased enzyme concentration, clear evidence for demethylation of H1.4K26me2 was observed with KDM7B in addition to KDM7A (Fig. S12E), but no significant difference was observed between the peptide with and without methylation at H1.4K21 (Fig. 2B). Thus, it appears unlikely that *N* ^ε^‐trimethylation at H1.4K21 promotes demethylation at H1.4K26 by KDM7B. These time courses, and similar ones with KDM7A, confirmed that the H1.4K26 peptides were significantly poorer substrates for the KDM7 subfamily than the H3K9 peptides (the weak demethylation efficiency observed for KDM7A/B precluded further kinetic analyses).

Studies then focused on determining the proficiency of H1.4K26 demethylation by the KDM4 enzymes. Specific activities of each enzyme with H1.4K26me3 were compared to those with H3K9me3, and more detailed studies were carried out with a representative KDM4 enzyme, KDM4A. ^1^H NMR time course analyses with KDM4A and the trimethylated H1.4K26me3 peptide confirmed time‐dependent demethylation of the Kme3 residue (Figs 2C, S13). The ^1^H NMR experiments also revealed concomitant turnover of 2OG into succinate (emergence of a singlet ^1^H resonance at δ_H_ 2.28 ppm, Fig. S13) that is consistent with the proposed demethylation mechanism 5.

Kinetic analyses were then undertaken using a fluorescence‐based formaldehyde dehydrogenase (FDH)‐coupled demethylation assay, which monitors the formation of NADH during FDH‐catalysed oxidation of formaldehyde 27. Initially, specific activities for each KDM4 enzyme with either H1.4K26me3 or H3K9me3 (each at 100 μm) were determined for comparison (Figs 2D, S14). These results revealed that in all cases demethylation of the two different marks was roughly comparable.

Michaelis–Menten kinetics were then conducted with KDM4A. Samples containing KDM4A, 2OG, ascorbate, H1.4K26me3 peptide, NAD^+^ and FDH were analysed over time and kinetic parameters were determined by recording the initial reaction rates of NADH production (corresponding to enzymatic production of formaldehyde) at varying peptide concentration. Similar experiments were carried out with H3K9me3 and H3K36me3 substrate peptides and the obtained values compared (Table 2). The experiments revealed that the H1.4K26me3 and H3K9me3 peptides are similarly efficient substrates of KDM4A, giving similar *K*_M_ (32.3 ± 6.4 μm and 25.5 ± 4.9 μm respectively) and *k*_*cat*_ (0.32 ± 0.049 s^−1^ and 0.31 ± 0.045 s^−1^ respectively) values. Demethylation of the H3K36me3 peptide was the least efficient, with higher *K*_M_ (66.8 ± 5.4 μm) and lower *k*_*cat*_ (0.12 ± 0.004 s^−1^) values than with the other two peptides.

**Table 2:** Kinetic parameters for demethylation of H1.4 peptides by recombinant human KDM4A (1 μm) measured using a formaldehyde dehydrogenase‐coupled demethylation assay 27. Data show the mean and standard deviation of three independent replicates. See Materials and methods for complete assay conditions

	*K*_M_/μm	*k*_cat_/s^−1^	*k*_cat_/*K*_M_
H3(1–15)K9me3	25.5 ± 4.9	0.31 ± 0.045	0.0124
H1.4(18–32)K26me3	32.3 ± 6.4	0.32 ± 0.049	0.0098
H3(29–43)K36me3	66.8 ± 5.4	0.12 ± 0.004	0.0019

**Figure 3:**
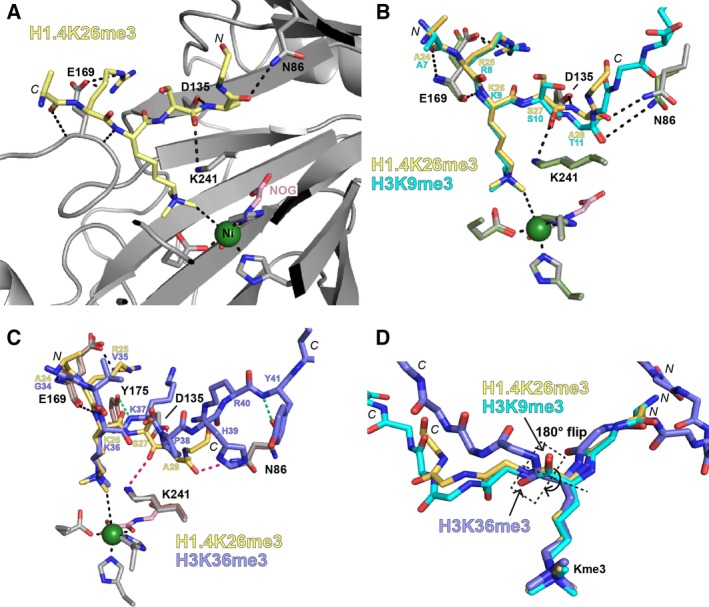
Binding mode of H1.4K26me3 to KDM4A. The figure shows views from (A) an X‐ray crystal structure of KDM4A bound to nickel (green, substitute for iron), NOG (pink, a 2OG mimetic inhibitor) and an H1.4(18–32)K26me3 peptide (yellow), alone, or overlaid with: (B) a structure of KDM4A bound to H3(7–14)K9me3 peptide (cyan, PDB ID http://www.rcsb.org/pdb/search/structidSearch.do?structureId=2OQ6) 26, and (C) a structure of KDM4A bound to an H3K36me3 peptide (violet, PDB ID http://www.rcsb.org/pdb/search/structidSearch.do?structureId=2P5B) 47. Residues from KDM4A in the H1.4K26 structure are shown as pale grey sticks, those from KDM4A in the H3K9 structure are shown as pale green sticks and those from KDM4A in the H3K36 structure are shown as pale pink (A–C). Some of the key interactions between KDM4A and the H1.4K26me3 peptide are shown by black dashes. Interactions between the H1.4K26me3 peptide and KDM4A conserved in the H3K9me3 peptide structure, but not in the H3K36me3 peptide structure are marked as pink dashes. Interactions only observed in the H3K36me3 peptide structure are shown as green dashes. (D) Overlay of the peptide backbones (and Kme3 side chain) of each peptide. The amide flip between the H3K36 peptide, and the H3K9 and H1.4K26 peptides, is highlighted with a dashed black box.

Crystallographic analyses were undertaken to investigate the binding mode of trimethylated H1.4K26 in the KDM4A active site. An X‐ray crystal structure of a KDM4A:Ni(II):*N*‐oxalylglycine:H1.4K26me3 peptide complex was solved to a resolution of 1.98 Å (Note: Ni(II) was used for crystallisation in place of the catalytically relevant Fe(II) due to its oxidative stability; *N*‐oxalylglycine (NOG) is a 2OG mimetic inhibitor) 46. Density was observed for H1.4 residues 24–29. Refinement revealed the H1.4K26me3 peptide bound in a cleft along the surface of KDM4A with the methylated K26 residue protruding towards the active site‐bound metal centre (Figs 3A, S1), positioning the carbon of the methyl group ~4 Å from the active site metal, apparently productively poised for enzymatic hydroxylation and subsequent demethylation.

Comparison of the H1.4K26me3 complex structure with those for a KDM4A:Ni(II):NOG:H3K9me3 peptide complex (PDB: http://www.rcsb.org/pdb/search/structidSearch.do?structureId=2OQ6 26, Fig. 3B) and a KDM4A:Ni(II):NOG:H3K36me3 peptide complex (PDB: http://www.rcsb.org/pdb/search/structidSearch.do?structureId=2P5B 47, Fig. 3C) reveals that all three peptides bind with the side chain of the trimethyllysine residue in the same orientation. All three peptides have the same N*‐* to C*‐* directionality through the active site. The two residues N‐terminal to the trimethyllysine residue (A24, R25 in the H1.4 peptide, A7, R8 in the H3K9 peptide and G34, V35 in the H3K36 peptide) adopt a similar conformation/orientation in all three structures, with backbone hydrogen bonds between KDM4A E169 and the peptide residues at the −1 and −2 positions (Fig. 3B/C). However, differences arise in the conformations of the C‐terminal regions of the peptides. The H3K9me3 and H1.4K26me3 peptides manifest similar conformations/orientations for the three residues C‐terminal to the target trimethyllysine residue, sharing conserved interactions with KDM4A K241, D135 and N86 (black dashes, Fig. 3B). By contrast with the H3K9me_n_ and H1.4K26me3 substrate structures, the peptide backbone of H3K36 adopts a more ‘bent’ conformation, likely enforced by the presence of a proline (H3P38) in its sequence. Thus, in the K36 peptide, the backbone carbonyl of the H3K36 to K37 amide bond is ‘flipped’ ~180° compared to the analogous amide in the other two substrates (Fig. 3D). Unlike the H3K9 and H1.4K26 peptides (pink dashes, Fig. 3C), the H3K36 peptide displays no interactions with either KDM4A K241 or D135, nor with the side‐chain of N86. Instead, the backbone –NH of the H3K36‐K37 amide link interacts with the phenol of Y175 (green dashes, Fig. 3C).

Overall, the crystallographic analyses reveal the H1.4K26me3 substrate adopts a binding mode more similar to that of H3K9me3 than H3K36me3. This common binding geometry is supportive of their similar demethylation efficiencies.

## Discussion

Though lysyl methylations have been reported on linker histones 10, 45, the processes that both regulate these methylation levels and contribute to their functional roles are largely undefined. Demethylation of methylated lysine residues at K26 of linker H1.4 has been reported to be catalysed by the KDM4 subfamily of JmjC KDMs in cells 15, 43. In previous studies on H1.4, no activity was observed for the KDM3 or KDM6 subfamilies; however, the KDM7 subfamily, which, like the KDM4s, also act at H3K9 had not been tested for activity with H1.4 29, 48, 49. Our results demonstrate that at least in the context of histone fragment peptides, recombinantly produced members of both the human KDM4 and KDM7 subfamilies of JmjC KDMs are capable of catalysing demethylation of methylated lysine residues at K26 of linker H1.4. Demethylation of dimethylated H1.4K26 by KDM7A and KDM7B appears considerably less efficient than the well‐characterised H3K9me2 substrates for these enzymes, possibly precluding any biological relevance 29, 42, 44. However, kinetic analyses with KDM4A reveal that demethylation of the H1.4K26me3 peptide and the well‐characterised KDM4 H3K9me3 substrate are comparably efficient, whereas the H3K36me3 substrate is less efficiently demethylated than either H3K9me3 or H1.4K26me3 under the conditions tested 23, 24, 50, 51.

Crystallographic analyses of H1.4K26me3 bound to KDM4A imply very similar binding modes for the H1.4K26me3 and an H3K9me3 peptides 26, 47, consistent with the kinetic studies which imply that they are substrates of approximately equal efficiency. Importantly, the H3K36me3 peptide, which at least *in vitro* is a less efficient substrate for KDM4A‐C (and not a substrate at all for KDM4D/E) than either H3K9me3 or H1.4K26me3, binds differently at the KDM4A active site 23, 47, 50. One clear difference is in the binding mode of the amide bond on the C‐terminal side of the *N*‐methylated substrate lysine for H3K9/H1.4K26 versus H3K36 (Fig. 3). Although how this observation relates to the different binding/catalytic efficiencies of the different peptide sequences is difficult to dissect, it suggests that subtle differences in binding conformation may have substantial effects on catalytic efficiency, an observation relevant to functional assignment work on JmjC KDMs 50, 52. Similar subtleties have been observed for chromatin reader domains; in some cases they bind similarly to the closely related H3K9 and H1.4K26 sequences (e.g. HP1) 17, whilst in others binding is only observed to one or the other (e.g. ankyrin repeats of G9A) 43.

The combined biochemical and structural studies thus identify methylated H1.4K26 peptides as substrates for JmjC KDMs with broadly comparable demethylation efficiencies to those reported for KDM4 substrates on core histones 23, 24, 50, 51. Given this potential for activity at H1.4, it is of interest to further investigate the consequence of methylation at this position *in vivo*, in particular in the context of ongoing medicinal chemistry studies targeting the KDM4 subfamily as a treatment for disease 7, 53. The methylation status of H1.4K26 is reported to regulate binding of HP1 and other chromodomain proteins 16, 17, which are associated with formation of heterochromatin. Thus, the reversal of this methylation by KDM4 enzymes in cells may well have direct effects on chromatin compaction and gene expression. It remains unclear whether a complex and dynamic ‘histone code’ akin to that proposed for the H3 N‐terminal tail, exists for H1. The dynamic modulation of PTMs would be a likely requirement for this. Regulation of PTMs on H1.4 has already been demonstrated to play important roles in gene regulation, as in the case of acetylation of H1.4K34, which may help recruit the general transcription factor IID through its bromodomain, and which increases the dynamic exchange of H1 54, or citrullination of H1.2R54, which results in histone eviction and chromatin decondensation 55. The ready demethylation of H1.4K26 as catalysed by the KDM4 demethylase family argues that this might also be the case for H1.4K26 methylation, at least for H1.4K26me3 12. Overall, we hope that our findings will stimulate further cellular work to characterise the full scope of JmjC KDM substrate selectivity and will be informative for ongoing studies with JmjC KDM inhibitors.

## Author contributions

LJW, RJH, AK and CJS conceived the idea. AK and CJS supervised the study. LJW, RJH, YZ, JB and RS conducted experiments and analysed the results. RC carried out the crystallography. LJW, RJH and CJS wrote the manuscript. All authors critically reviewed the manuscript.

## Supporting information


**Fig. S1.** Stereoview from a KDM4A.Ni.NOG.H1.4(18‐32)K26me3 crystal structure.
**Fig. S2.** KDM4E catalyses lysine demethylation at H1.4K26.
**Fig. S3.** KDM2A does not catalyse lysine demethylation at H1.4K26 under the tested conditions.
**Fig. S4.** KDM3A does not catalyse lysine demethylation at H1.4K26 under the tested conditions.
**Fig. S5.** KDM5C does not catalyse lysine demethylation at H1.4K26 under the tested conditions.
**Fig. S6**. KDM6B does not catalyse lysine demethylation at H1.4K26 under the tested conditions.
**Fig. S7.** KDM7A catalyses lysine demethylation at H1.4K26.
**Fig. S8.** KDM4A catalyses lysine demethylation at H1.4K26.
**Fig. S9.** KDM4B catalyses lysine demethylation at H1.4K26.
**Fig. S10.** KDM4C catalyses lysine demethylation at H1.4K26.
**Fig. S11.** KDM4D catalyses lysine demethylation at H1.4K26.
**Fig. S12.** PHF8/KDM7B only catalyses lysine demethylation at H1.4K26 at high concentration.
**Fig. S13.** Analysis of KDM4A demethylation by ^1^H NMR.
**Fig. S14.** Specific activity determination for KDM4 enzymes.
**Table S1.** Peptide sequences used in this study.
**Table S2.** Crystallographic data processing and refinement statistics.
