# Supplementary material for: Mechanistic and structural studies of KDM‐catalysed demethylation of histone 1 isotype 4 at lysine 26
Source: FEBS Lett. 2018 Sep 14;592(19):3264–73. doi: 10.1002/1873-3468.13231 (PMC6220849; doi:10.1002/1873-3468.13231)
Supplement: Supplementary file 1 — Fig. S1. Stereoview from a KDM4A.Ni.NOG.H1.4(18‐32)K26me3 crystal structure. Fig. S2. KDM4E catalyses lysine demethylation at H1.4K26. Fig. S3. KDM2A does not catalyse lysine demethylation at H1.4K26 under the tested conditions. Fig. S4. KDM3A does not catalyse lysine demethylation at H1.4K26 under the tested conditions. Fig. S5. KDM5C does not catalyse lysine demethylation at H1.4K26 under the tested conditions. Fig. S6. KDM6B does not catalyse lysine demethylation at H1.4K26 under the tested conditions. Fig. S7. KDM7A catalyses lysine demethylation at H1.4K26. Fig. S8. KDM4A catalyses lysine demethylation at H1.4K26. Fig. S9. KDM4B catalyses lysine demethylation at H1.4K26. Fig. S10. KDM4C catalyses lysine demethylation at H1.4K26. Fig. S11. KDM4D catalyses lysine demethylation at H1.4K26. Fig. S12. PHF8/KDM7B only catalyses lysine demethylation at H1.4K26 at high concentration. Fig. S13. Analysis of KDM4A demethylation by 1H NMR. Fig. S14. Specific activity determination for KDM4 enzymes. Table S1. Peptide sequences used in this study. Table S2. Crystallographic data processing and refinement statistics. [file FEB2-592-3264-s001.docx]

**Mechanistic and Structural Studies of KDM-Catalysed Demethylation of Histone 1 Isotype 4 at Lysine 26**

Louise J. Walport^1^, Richard J. Hopkinson^1,2^, Rasheduzzaman Chowdhury^1^, Yijia Zhang^1^, Joanna Bonnici^1^, Rachel Schiller^1^, Akane Kawamura^1,3^, and Christopher J. Schofield^1,#^

﻿

^1^Department of Chemistry, Chemistry Research Laboratory, University of Oxford, Mansfield Road, Oxford OX1 3TA, United Kingdom.

^2^Leicester Institute of Structural and Chemical Biology and Department of Chemistry, University of Leicester, Henry Wellcome Building, Lancaster Road, Leicester, United Kingdom.

^3^Division of Cardiovascular Medicine, Radcliffe Department of Medicine, Wellcome Trust Centre for Human Genetics, Roosevelt Drive, Oxford OX3 7BN, United Kingdom.

^#^Correspondence and requests for materials should be addressed to CJS (email: [christopher.schofield@chem.ox.ac.uk](mailto:christopher.schofield@chem.ox.ac.uk), tel: +44(0)1865 285 006).

**
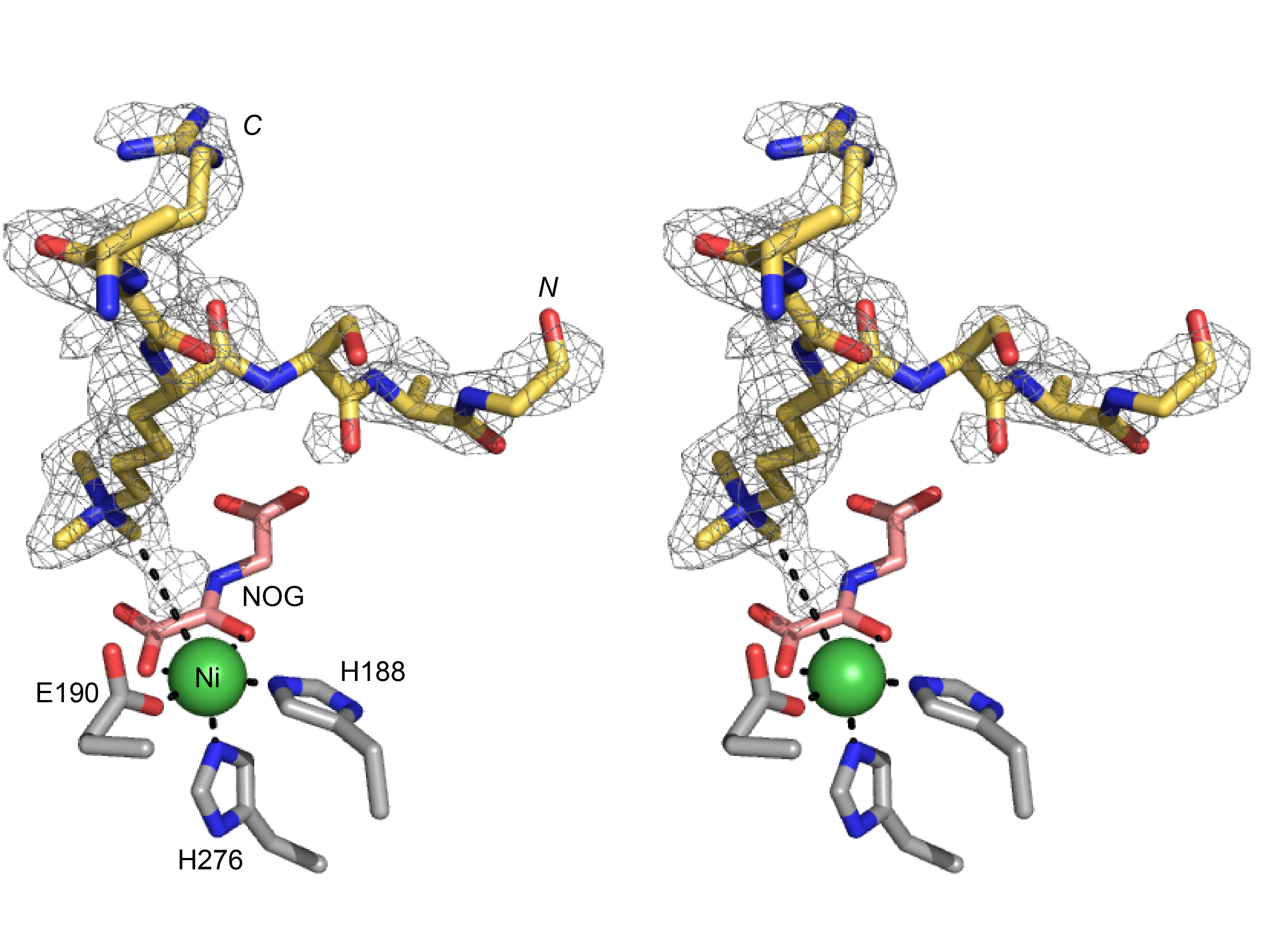
**

**Figure S1 Stereoview from a KDM4A.Ni.NOG.H1.4(18-32)K26me3 crystal structure.** ﻿Stereoview from PDB ID 6H8P showing the Fo-Fc OMIT map contoured to 3σ around H1.4(18-32)K26me3 peptide residues, shown in yellow. Ni (substituting for Fe) is shown in green, NOG (a 2OG mimetic inhibitor) is shown in pink and metal-binding residues are shown in grey.


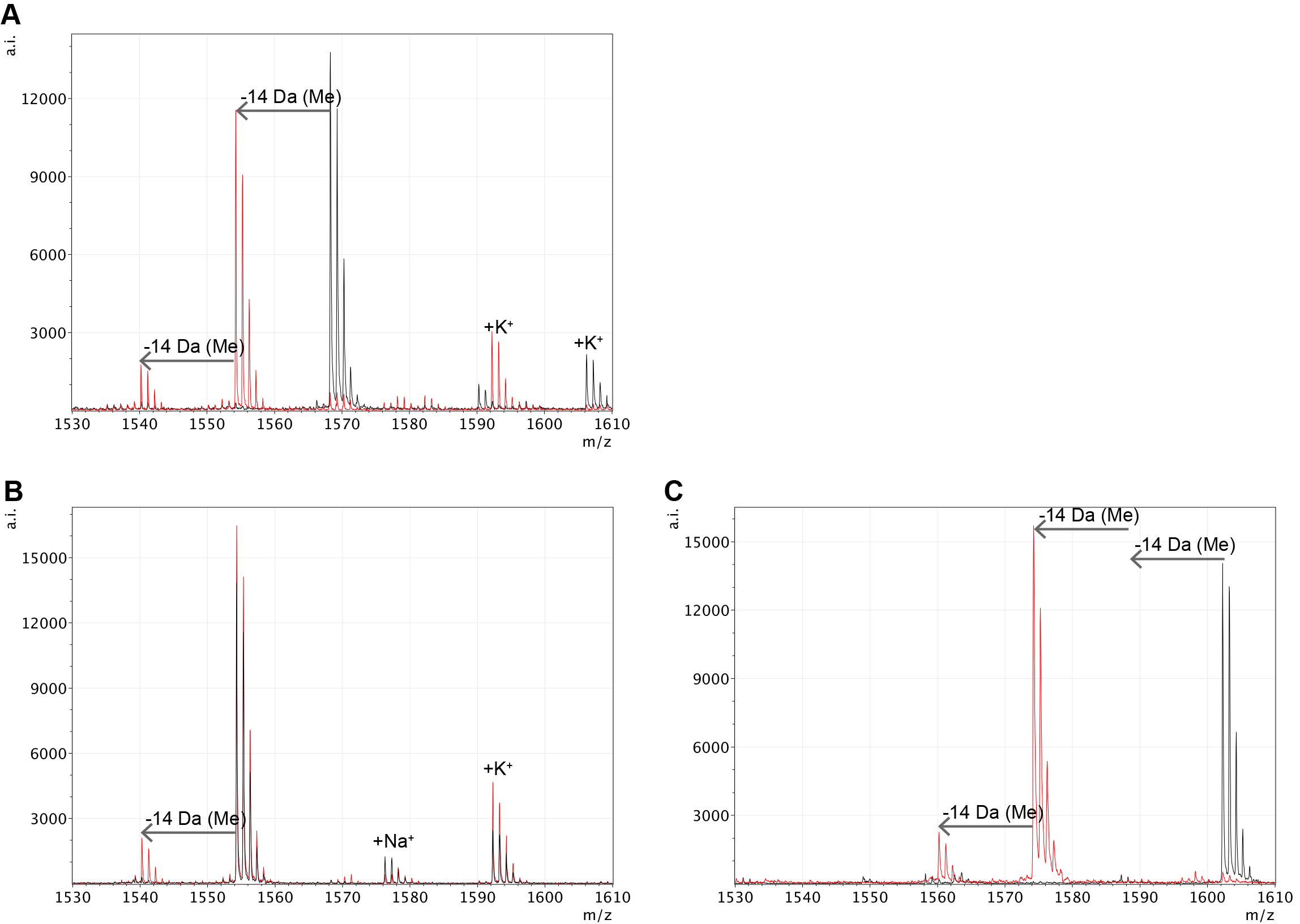


**Figure S2 KDM4E catalyses lysine demethylation at H1.4K26.** MALDI-TOF MS spectra of demethylation reactions. The red spectra show reactions with KDM4E; the black spectra no enzyme controls. **A)** H1.4(18-32)K26me2; **B)** H1.4(18-32)K26me1; **C)** H3(1-15)K9me3. The assay for H1.4(18-32)K26me3 is shown in the main text.

**
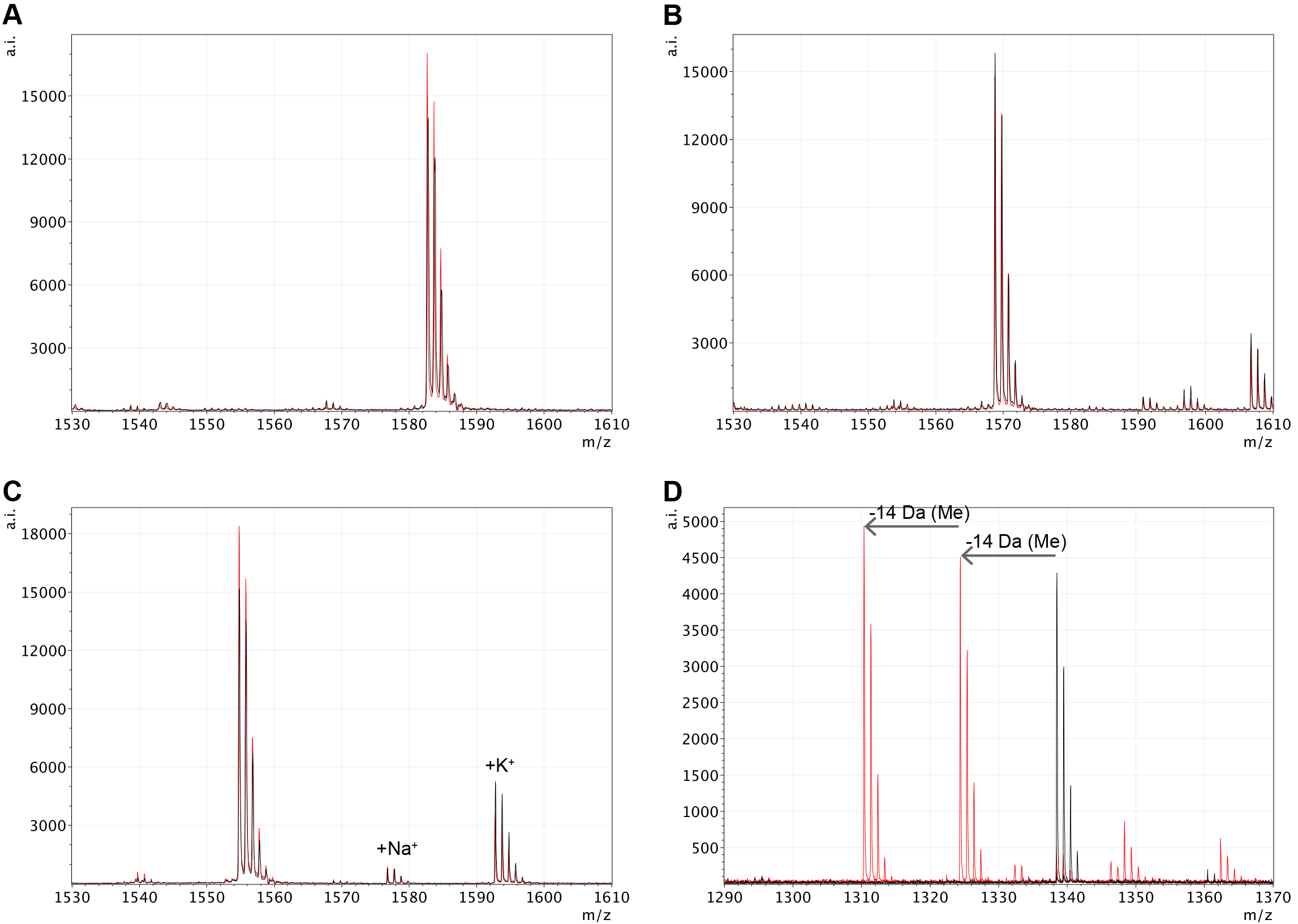
**

**Figure S3 KDM2A does not catalyse lysine demethylation at H1.4K26 under the tested conditions.** MALDI-TOF MS spectra of demethylation reactions. The red spectra show reactions with KDM2A; the black spectra no enzyme controls. **A)** H1.4(18-32)K26me3; **B)** H1.4(18-32)K26me2; **C)** H1.4(18-32)K26me1; **D)** H3(30-41)K36me2.


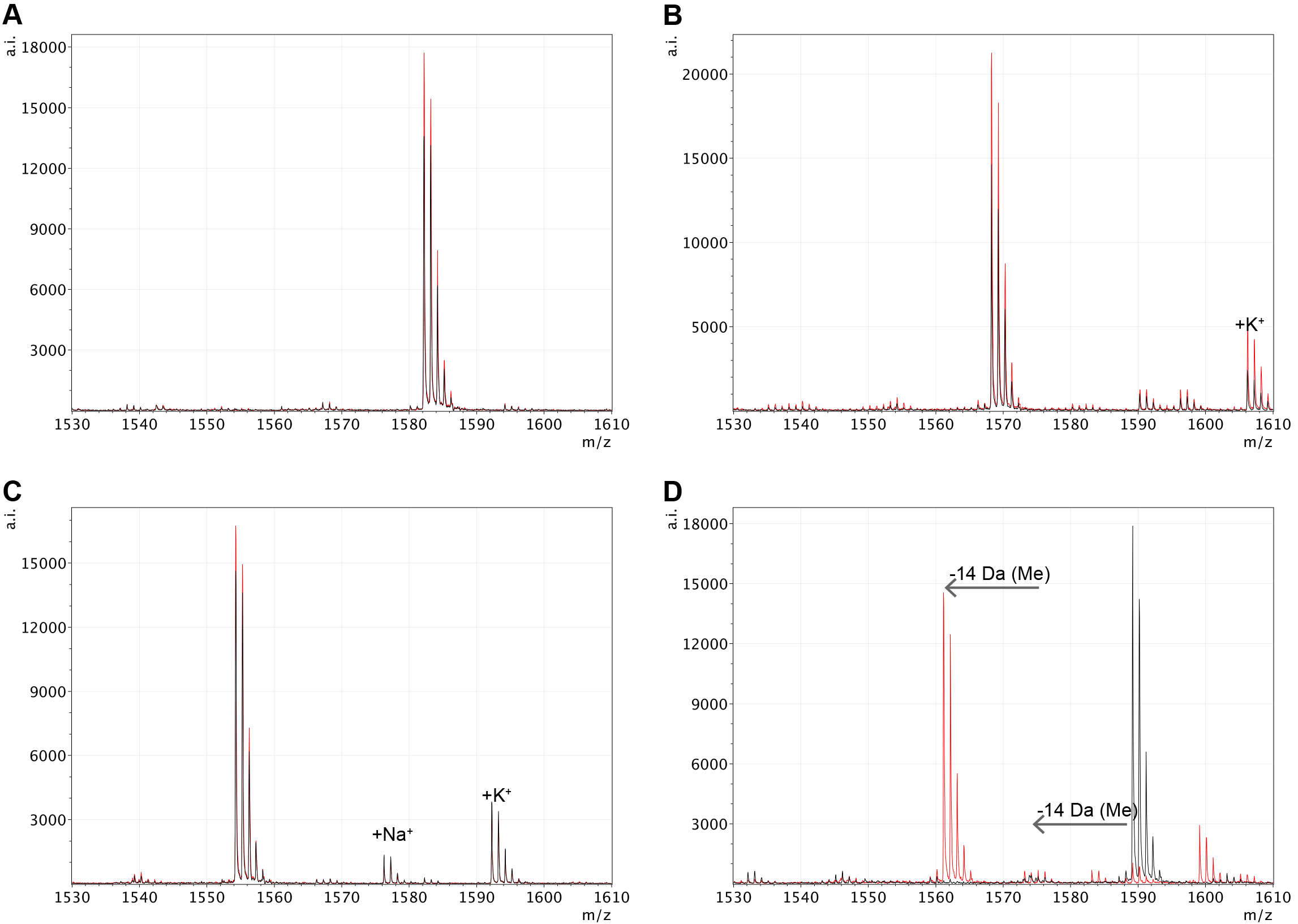


**Figure S4 KDM3A does not catalyse lysine demethylation at H1.4K26 under the tested conditions.** MALDI-TOF MS spectra of demethylation reactions. The red spectra show reactions with KDM3A; the black spectra no enzyme controls. **A)** H1.4(18-32)K26me3; **B)** H1.4(18-32)K26me2; **C)** H1.4(18-32)K26me1; **D)** H3(1-15)K9me2.


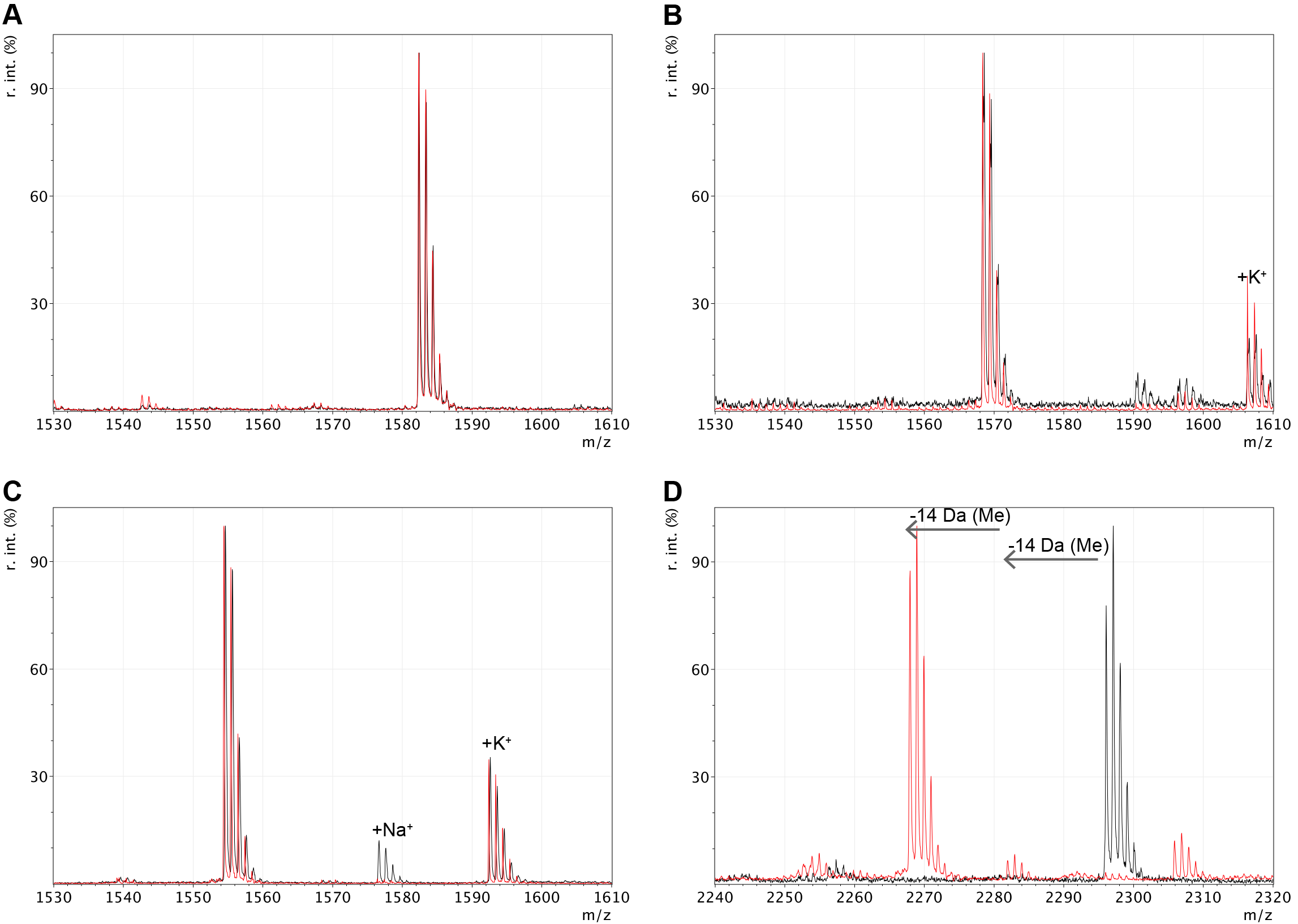


**Figure S5 KDM5C does not catalyse lysine demethylation at H1.4K26 under the tested conditions.** MALDI-TOF MS spectra of demethylation reactions. The red spectra show reactions with KDM5C; the black spectra no enzyme controls. **A)** H1.4(18-32)K26me3; **B)** H1.4(18-32)K26me2; **C)** H1.4(18-32)K26me1; **D)** H3(1-21)K4me3.


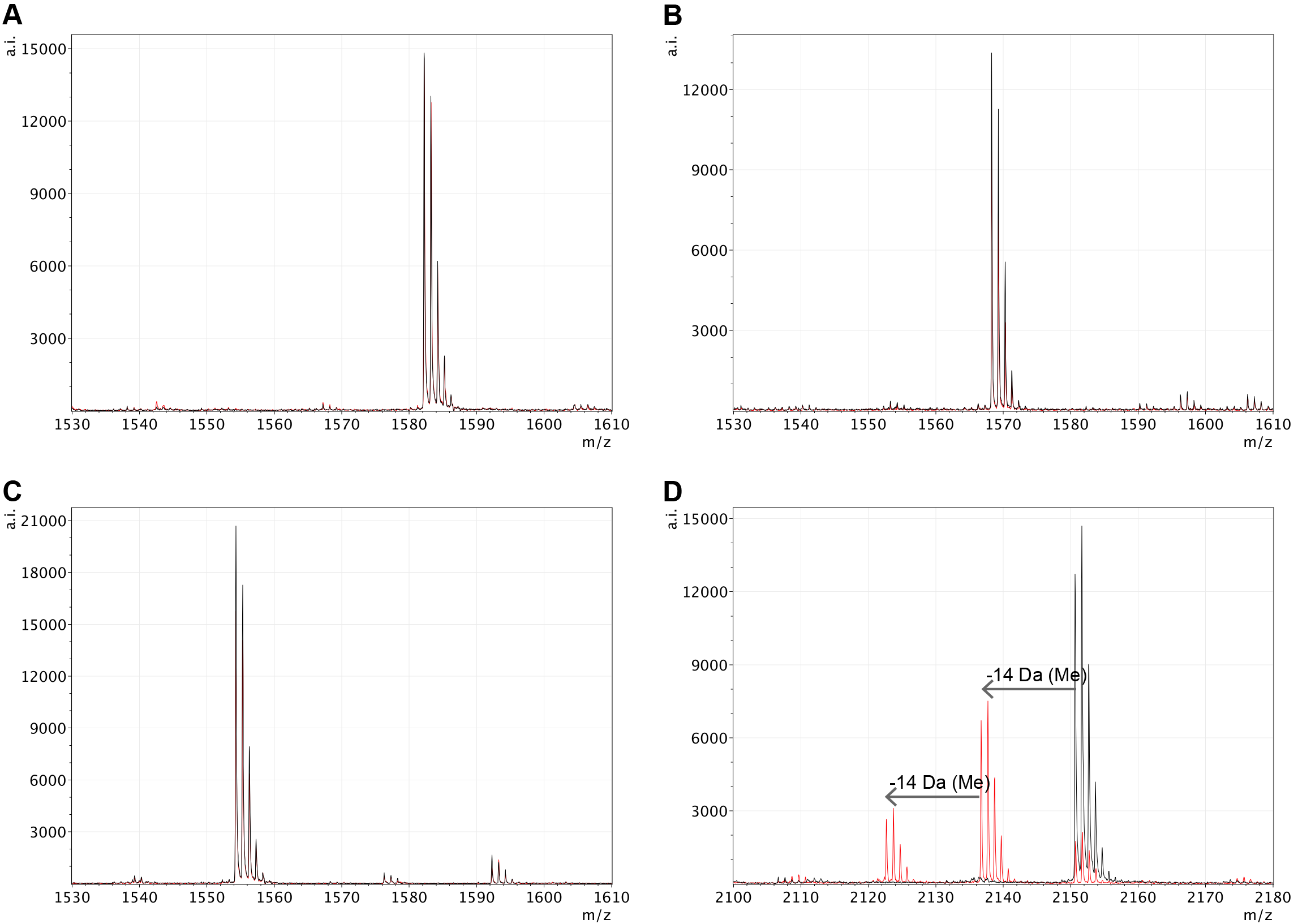


**Figure S6 KDM6B does not catalyse lysine demethylation at H1.4K26 under the tested conditions.** MALDI-TOF MS spectra of demethylation reactions. The red spectra show reactions with KDM6B; the black spectra no enzyme controls. **A)** H1.4(18-32)K26me3; **B)** H1.4(18-32)K26me2; **C)** H1.4(18-32)K26me1; **D)** H3(1-21)K27me3.


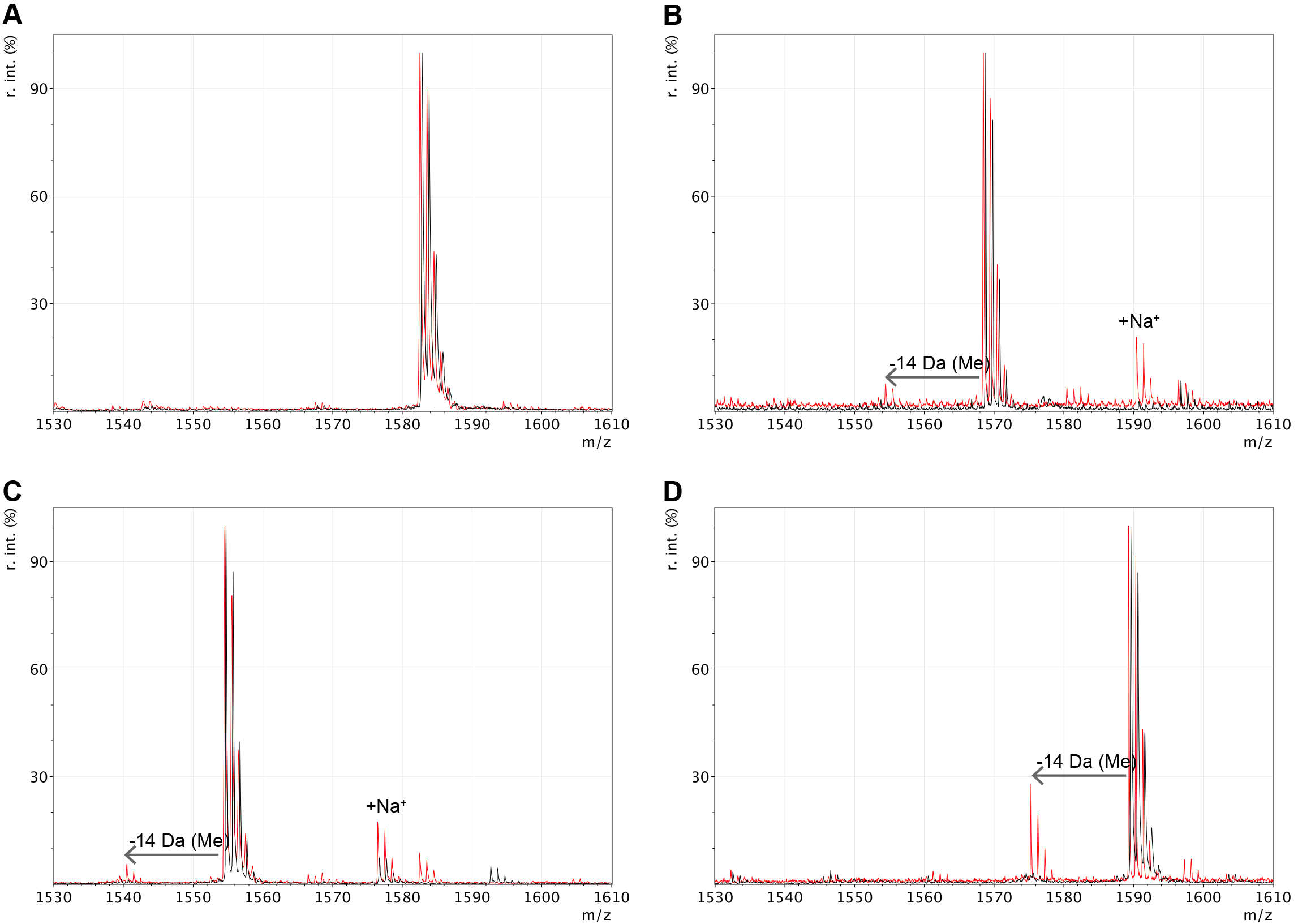


**Figure S7 KDM7A catalyses lysine demethylation at H1.4K26.** MALDI-TOF MS spectra of demethylation reactions. The red spectra show reactions with KDM7A; the black spectra show reactions quenched at 0 min. **A)** H1.4(18-32)K26me3; **B)** H1.4(18-32)K26me2; **C)** H1.4(18-32)K26me1; **D)** H3(1-21)K9me2.


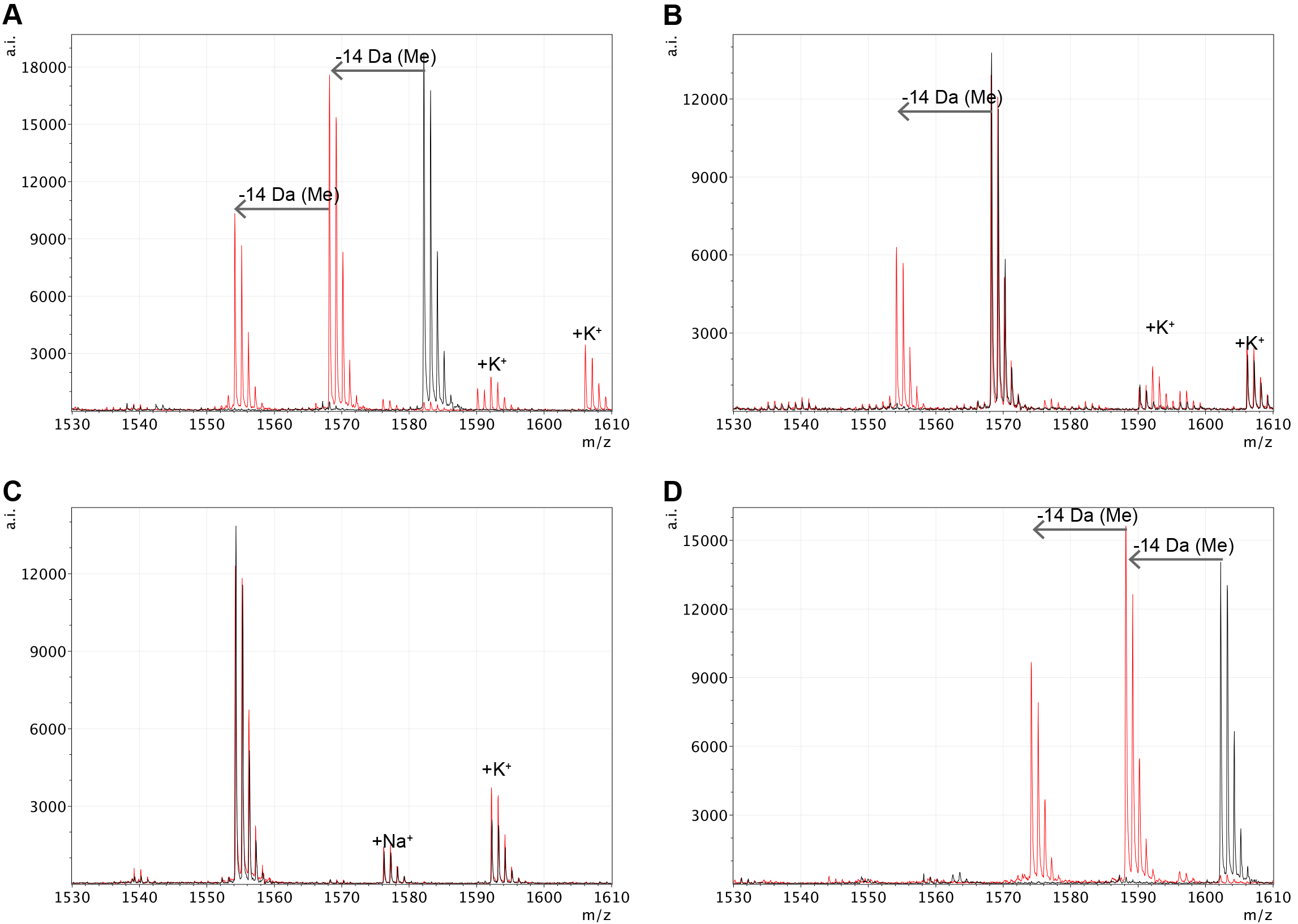


**Figure S8 KDM4A catalyses lysine demethylation at H1.4K26.** MALDI-TOF MS spectra of demethylation reactions. The red spectra show reactions with KDM4A; the black spectra no enzyme controls. **A)** H1.4(18-32)K26me3; **B)** H1.4(18-32)K26me2; **C)** H1.4(18-32)K26me1; **D)** H3(1-15)K9me3.


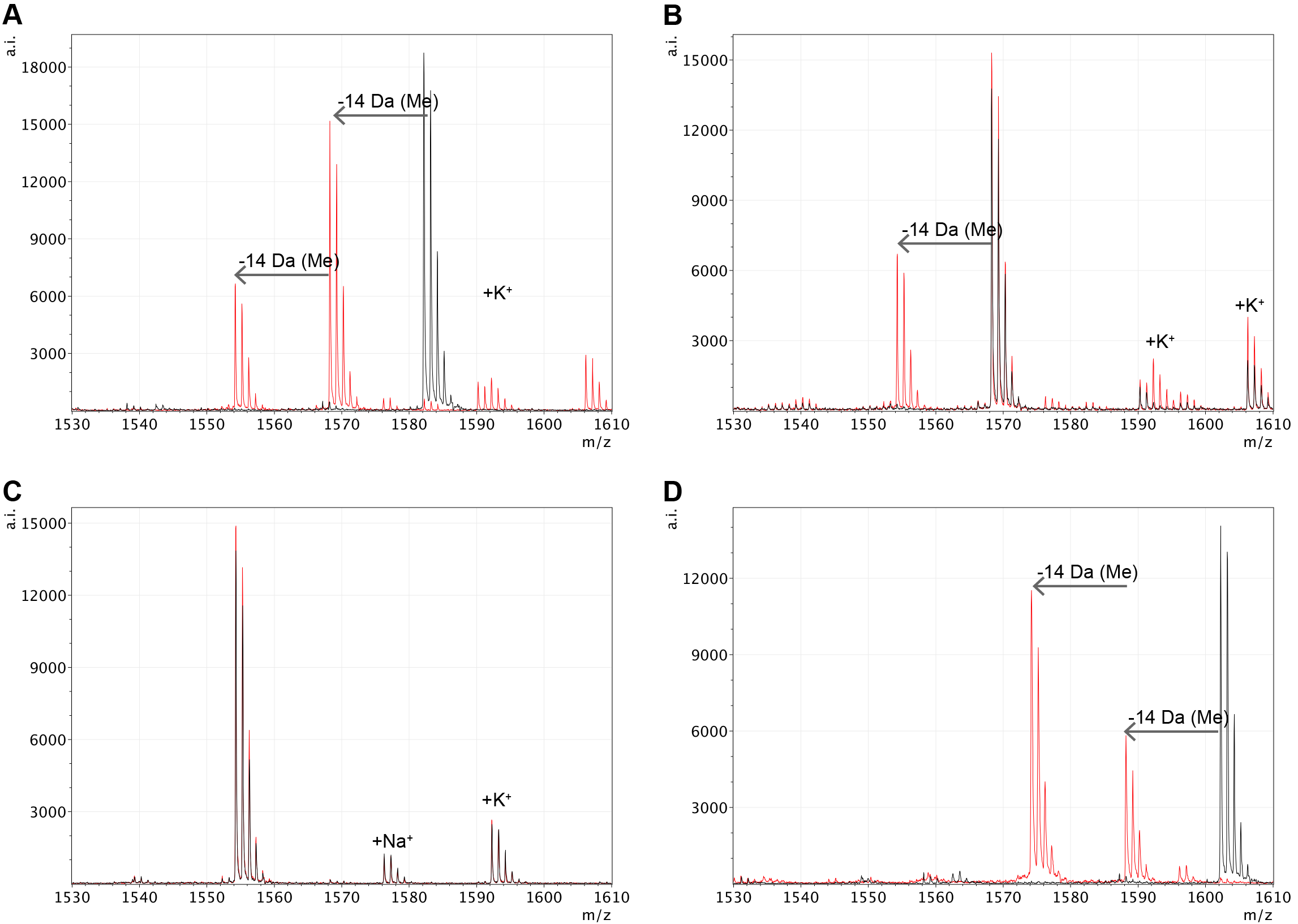


**Figure S9 KDM4B catalyses lysine demethylation at H1.4K26.** MALDI-TOF MS spectra of demethylation reactions. The red spectra show reactions with KDM4B; the black spectra no enzyme controls. **A)** H1.4(18-32)K26me3; **B)** H1.4(18-32)K26me2; **C)** H1.4(18-32)K26me1; **D)** H3(1-15)K9me3.


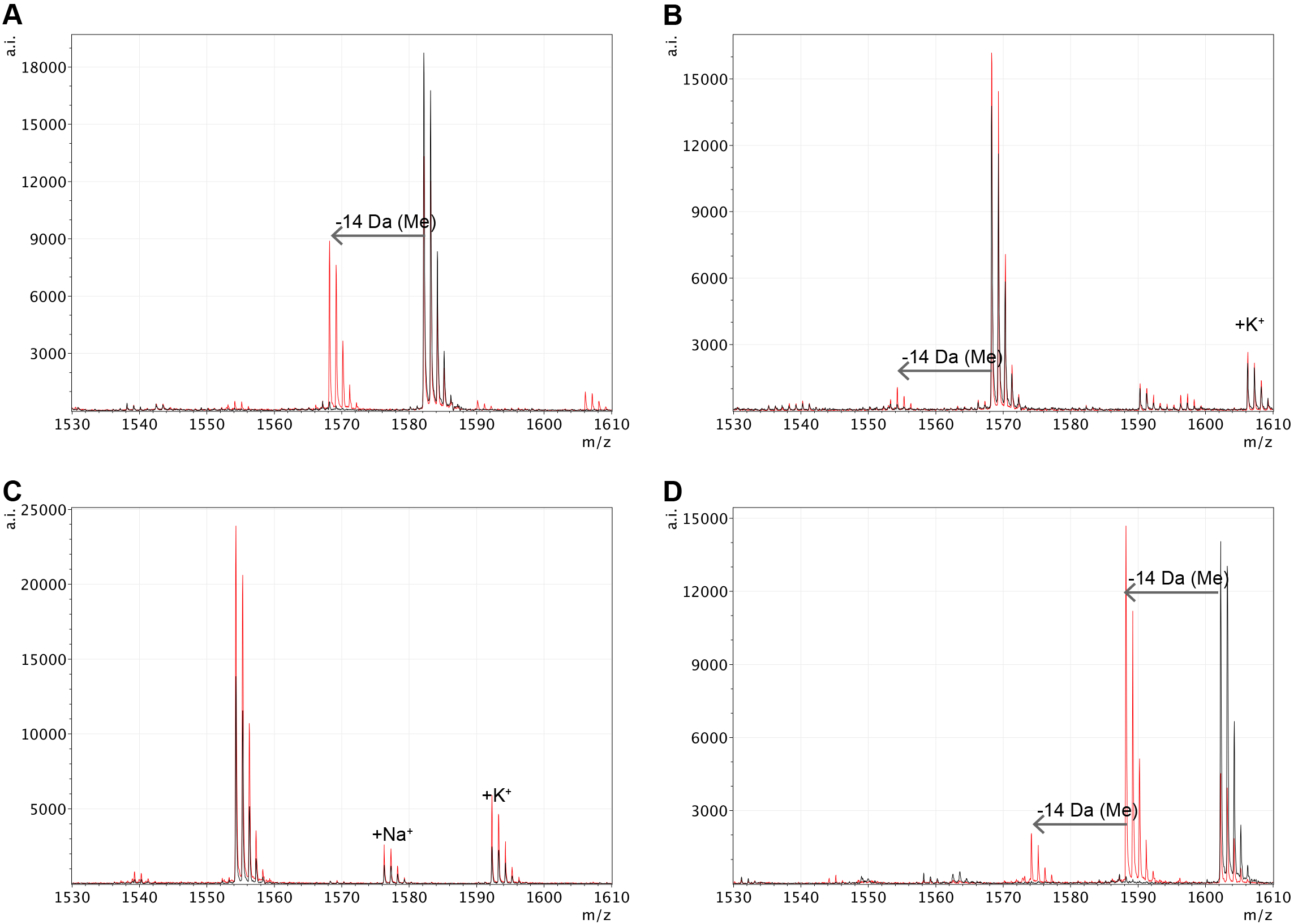


**Figure S10 KDM4C catalyses lysine demethylation at H1.4K26.** MALDI-TOF MS spectra of demethylation reactions. The red spectra show reactions with KDM4C; the black spectra no enzyme controls. **A)** H1.4(18-32)K26me3; **B)** H1.4(18-32)K26me2; **C)** H1.4(18-32)K26me1; **D)** H3(1-15)K9me3.


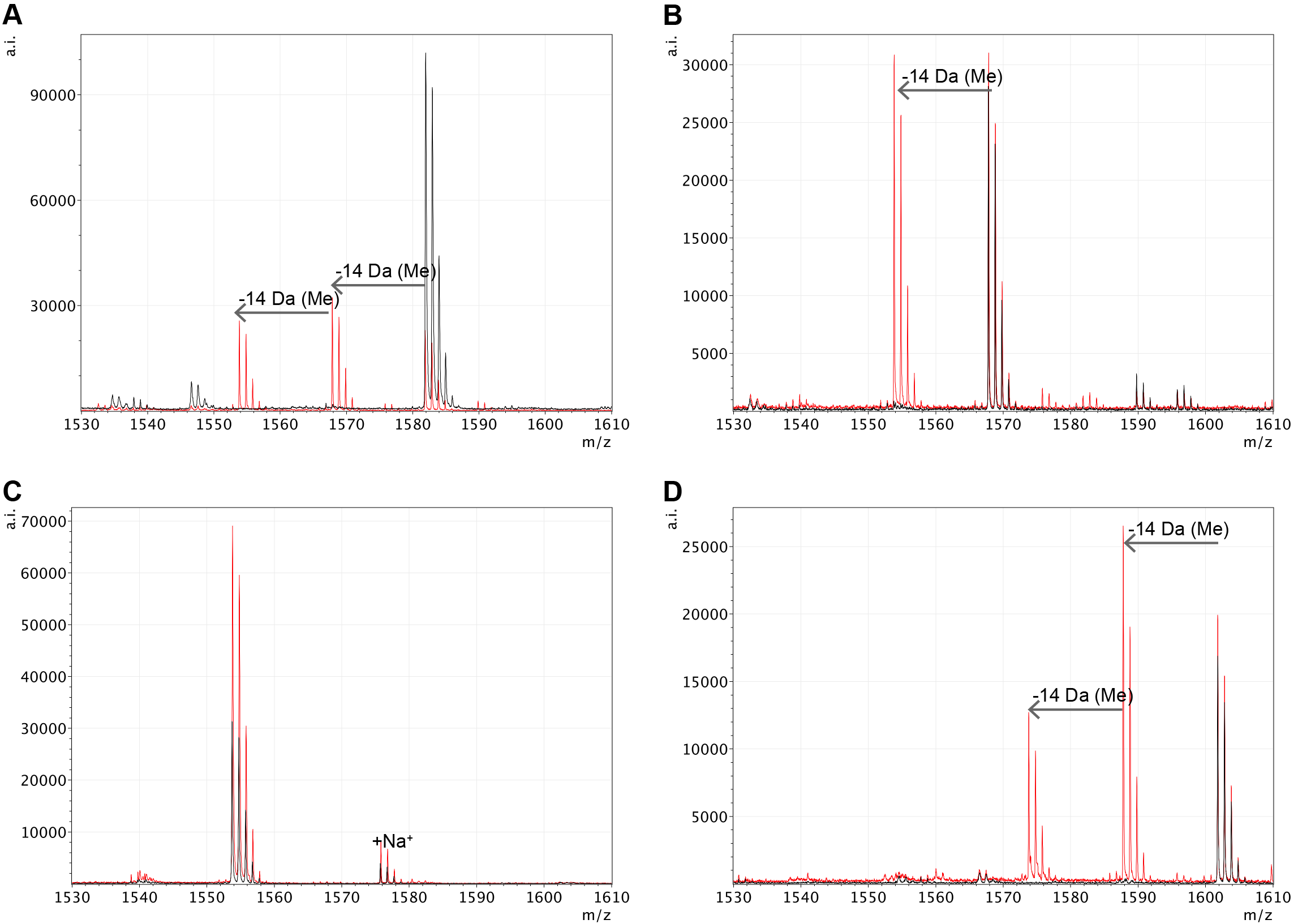


**Figure S11 KDM4D catalyses lysine demethylation at H1.4K26.** MALDI-TOF MS spectra of demethylation reactions. The red spectra show reactions with KDM4D; the black spectra no enzyme controls. **A)** H1.4(18-32)K26me3; **B)** H1.4(18-32)K26me2; **C)** H1.4(18-32)K26me1; **D)** H3(1-15)K9me3.


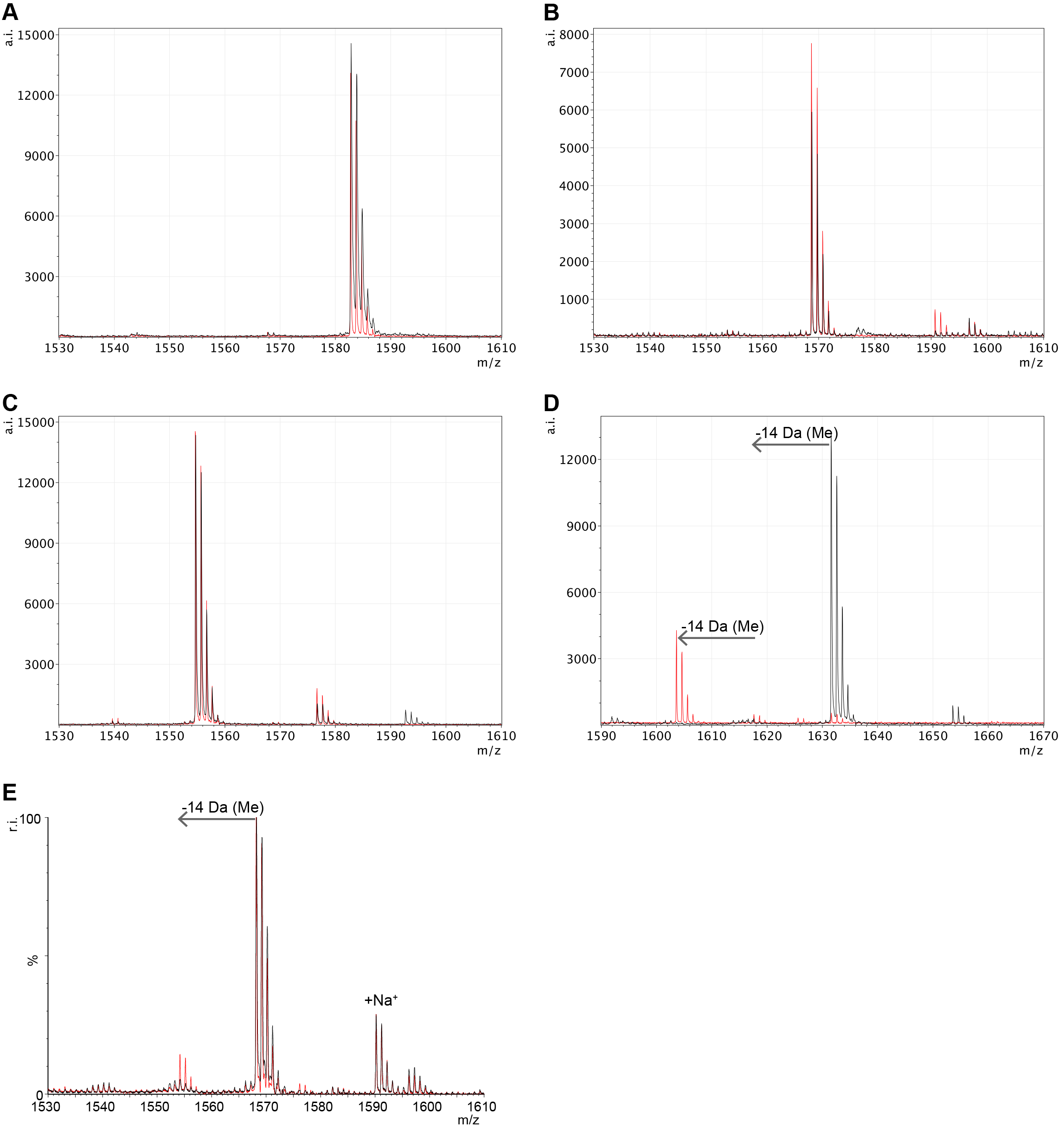


**Figure S12 PHF8/KDM7B only catalyses lysine demethylation at H1.4K26 at high concentration.** MALDI-TOF MS spectra of demethylation reactions. The red spectra show reactions with KDM7B (1 µM **A-D**, 5 µM **E**); the black spectra show reactions quenched at 0 min. **A)** H1.4(18-32)K26me3; **B)** H1.4(18-32)K26me2; **C)** H1.4(18-32)K26me1; **D)** H3(1-15)K4me3K9me2. **E)** Note that with a fivefold increased concentration of KDM7B (i.e. 5 µM) demethylation of H1.4(18-32)K26me2 is observed (Figure 2).


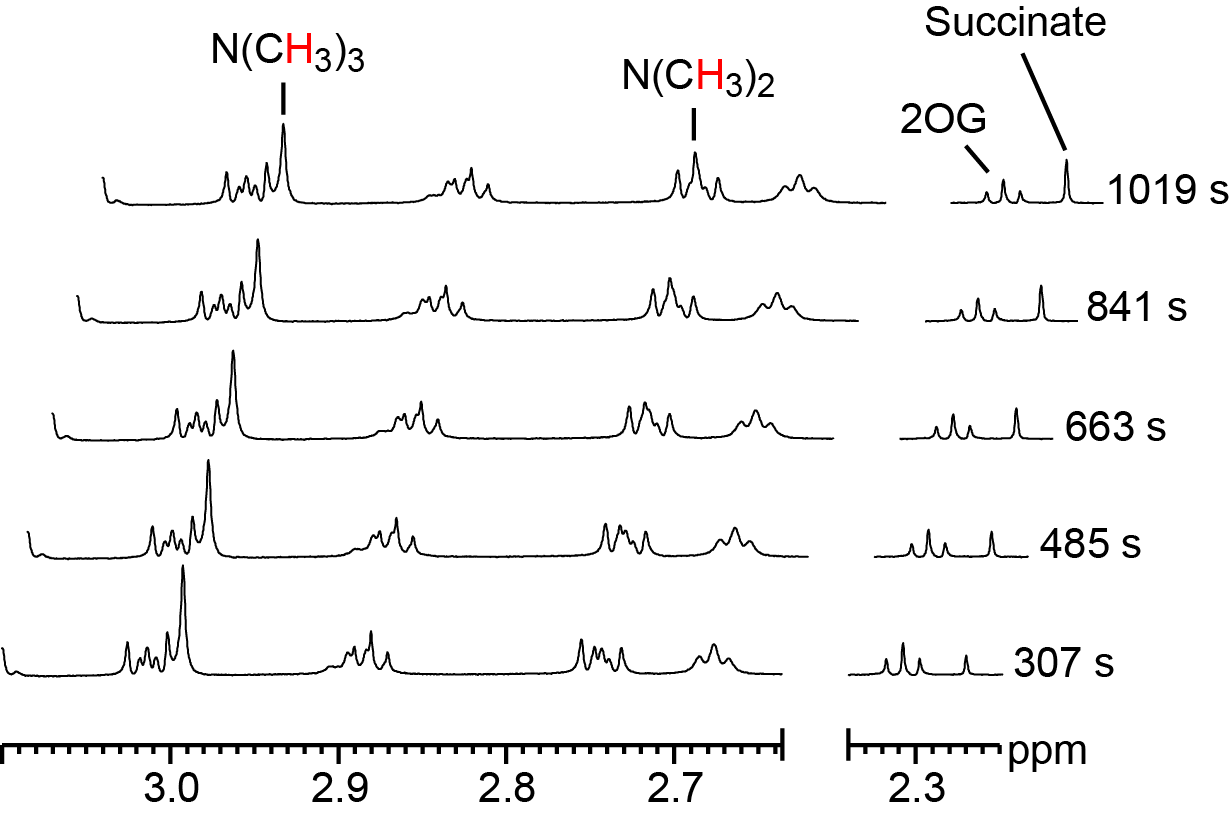


**Figure S13 Analysis of KDM4A demethylation by ^1^H NMR.** Time course experiments containing KDM4A (10 µM) and the H1.4K26me3 peptide (400 µM) were analysed by ^1^H NMR (700 MHz). Full conditions are given in the Methods section in the main text.


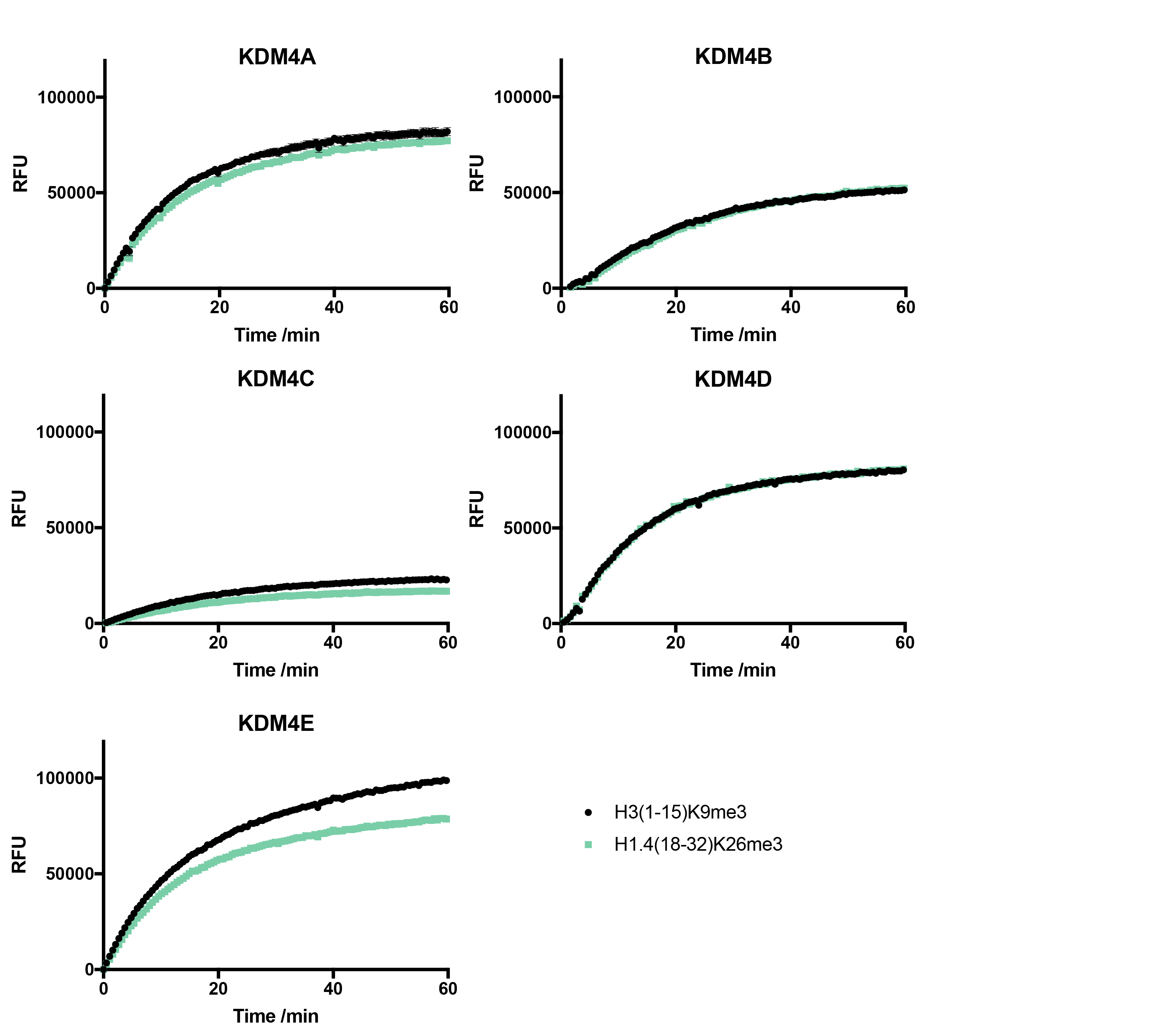


**Figure S14 Specific activity determination for KDM4 enzymes.** FDH experiments were conducted with each KDM4 (1 µM) and the H1.4K26me3 or H3K9me3 peptide (100 µM). Initial rates were used to calculate specific activities for each enzyme/peptide pair, for which the data is given in the main text. Full conditions are given in the Methods section in the main text.

**Supplementary Table 1.** Peptide sequences used in this study. All peptides were prepared as C-terminal amides. Peptides are observed as [M+H]^+^ in the MALDI-TOF MS.

| **Position** | **Sequence** | **Monoisotopic Mass /Da** |
| --- | --- | --- |
| H1.4K26me3 | TPVKKKAR-**Kme3**-SAGAAK | 1581 |
| H1.4K26me2 | TPVKKKAR-**Kme2**-SAGAAK | 1567 |
| H1.4K26me1 | TPVKKKAR-**Kme1**-SAGAAK | 1553 |
| H1.4K21me3K26me2 | TPV-Kme3-KKAR-**Kme2**-SAGAAK | 1609 |
| H1.4K21me3K26me1 | TPV-Kme3-KKAR-**Kme1**-SAGAAK | 1595 |
| H3K4me3 | ART-Kme3-QTARKSTGGKAPRKQLA | 2294 |
| H3K4me3K9me2 | ART-Kme3-QTAR-**Kme2**-STGGKA | 1631 |
| H3K9me2 | ARTKQTAR-**Kme2**-STGGKA | 1587 |
| H3K9me3 | ARTKQTAR-**Kme3**-STGGKA | 1602 |
| H3K27me3 | KQLATKAAR-**Kme3**-SAPSTG | 1656 |
| H3K36me3 | APATGGV-**Kme3**-KPHRYRP | 1675 |
| H3K36me2 | PATGGV-**Kme2**-KPHRY | 1337 |

**Table S2. Crystallographic data processing and refinement statistics**

| **Data Collection** |  |
| --- | --- |
| Beamline (Wavelength, Å) | Dls i04-1 (0.91741) |
| Detector | Pilatus 2M |
| Data processing | HKL2000 |
| Space Group | P21212 |
| Cell dimensions a,b,c (Å) | 100.722 |
|  | 150.146 |
|  | 57.601 |
| No. of molecules/ ASU | 2 |
| Resolution (Å) | 47.45 – 1.98 (2.05 – 1.98)* |
| No. of unique reflections | 60704 (5991)* |
| Completeness (%) | 99.1 (99.4)* |
| Redundancy | 8.1 (8.1)* |
| R_sym_ | 0.083 (0.937) |
| Mean I/σ(I) | 26.5 (2.5)* |
| Wilson *B* value (Å^2^) | 37.26 |
|  |  |
| **Refinement** |  |
| R_factor_ | 0.195 |
| R_free_ | 0.209 |
| R.m.s. deviation |  |
| Bond length, Å | 0.003 |
| Bond angle, ° | 0.680 |
|  |  |

*Highest resolution shell shown in parentheses.
